# Integrative analysis to screen novel pyroptosis-related LncRNAs for predicting clinical outcome of glioma and validation in tumor tissue

**DOI:** 10.18632/aging.204580

**Published:** 2023-03-13

**Authors:** Shuai Ma, Hongtao Zhao, Fang Wang, Lulu Peng, Heng Zhang, Zaibin Wang, Fan Jiang, Dongtao Zhang, Menglei Yin, Shupeng Li, Jiaming Huang, Zhan Liu, Shengzhong Tao

**Affiliations:** 1 Department of Neurosurgery, The Second Affiliated Hospital of Zhengzhou University, Zhengzhou 450053, China; 2 Department of Neurosurgery, Dalian Municipal Central Hospital Affiliated of Dalian Medical University, Dalian 116000, China; 3 Department of Neurosurgery, Cancer Center, Zhejiang Provincial People's Hospital, Affiliated People's Hospital, Hangzhou Medical College, Hangzhou, Zhejiang 310006, China; 4 Department of Neurosurgery, The Second Affiliated Hospital of Harbin Medical University, Harbin 150001, China

**Keywords:** pyroptosis-related LncRNA, tumor immune microenvironment, PRLPM riskscore, immunotherapy, chemotherapy

## Abstract

Background: Pyroptosis, also known as inflammatory necrosis, is a programmed cell death that manifests itself as a continuous swelling of cells until the cell membrane breaks, leading to the liberation of cellular contents, which triggers an intense inflammatory response. Pyroptosis might be a panacea for a variety of cancers, which include immunotherapy and chemotherapy-insensitive tumors such as glioma. Several findings have observed that long non-coding RNAs (lncRNAs) modulate the bio-behavior of tumor cells by binding to RNA, DNA and protein. Nevertheless, there are few studies reporting the effect of lncRNAs in pyroptosis processes in glioma.

Methods: The principal goal of this study was to identify pyroptosis-related lncRNAs (PRLs) utilizing bioinformatic algorithm and to apply PCR techniques for validation in human glioma tissues. The second goal was to establish a prognostic model for predicting the overall survival patients with glioma. Predict algorithm was used to construct prognosis model with good diagnostic precision for potential clinical translation.

Results: Noticeably, molecular subtypes categorized by the PRLs were not distinct from any previously published subtypes of glioma. The immune and mutation landscapes were obviously different from previous subtypes of glioma. Analysis of the sensitivity (IC50) of patients to 30 chemotherapeutic agents identified 22 agents as potential therapeutic agents for patients with low riskscores.

Conclusions: We established an exact prognostic model according to the expression profile of PRLs, which may facilitate the assessment of patient prognosis and treatment patterns and could be further applied to clinical.

## INTRODUCTION

Glioma is a frequent tumor that is the leading reason of cancer associated deaths and prone to chemo-resistance as one of the primary causes of treatment outcomes [1, 2]. Pyroptosis, an inflammatory cell death pathway, not only enhances the sensitivity of chemotherapy to tumors, but also efficiently induces anti-tumor immune activity in the body [3, 4]. Notably, pyroptosis may be a powerful weapon tool in the treatment of many chemo-resistant tumors, especially glioma. Due to the absence of efficient drugs for clinical treatment of gliomas, novel druggable targets are strongly needed. Therefore, pyroptosis is considered to be a “key player” in glioma, and is an effective tumor-killing process that can be targeted. Nevertheless, the regulatory mechanism and clinical significance of pyroptosis in tumors such as glioma are still confusing and need further investigation.

Previous researches have emphasized the effect of protein-coding genes in pyroptosis and confirmed a number of genes that affect pyroptosis sensitivity, including Gasdermins and Casp genes [2, 5]. Nevertheless, there are few reports on whether and how non-coding RNA regulate pyroptosis [6, 7]. Long non-coding RNAs (lncRNAs) are non-coding RNAs of more than 200 nucleotides [8]. Abundant studies have revealed that lncRNAs regulate the bio-behavior of glioma [9–11]. Several studies have demonstrated that lncRNAs modulate pyroptosis through a competitive endogenous RNA mechanism, acting as sponges on particular micro RNAs and attenuating inhibition of targeted mRNAs [7, 12, 13]. Additional findings have revealed that lncRNAs could also influence pyroptosis by directly binding to proteins or suppressing the translational process, suggesting the diverse effects of lncRNAs in the regulation of pyroptosis.

Thus, the validation of pyroptosis associated lncRNAs (PRLs) is essential to unravel the underlying mechanism of gliomagenesis and identify new therapeutic targets. In this study, PRLs were identified and a prognostic model according to PRLs matrix was constructed, which possessed high-level diagnostic precision and was associated with immune microenvironment and the mutation landscape.

## RESULTS

### Identification of prognostically significant PRLs in glioma patient samples

**Figure 1:**
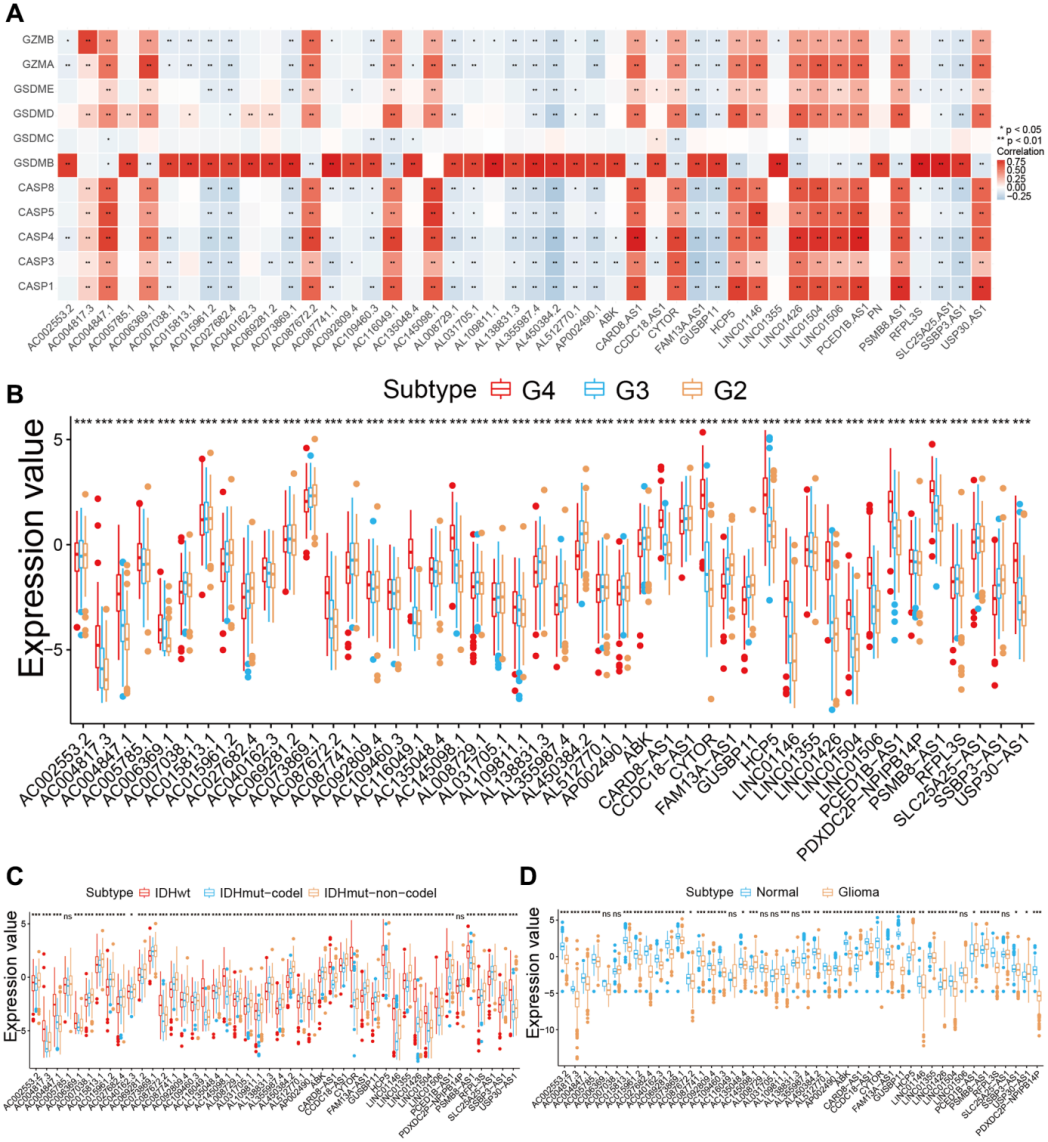
**Construction and validation of the 46 pyroptosis-related lncRNA (PRLs) signature in Glioma patients.** (**A**) Correlations between PRLs and pyroptosis regulator for glioma (Pearson test). (**B**) The expression of 46 PRLs among grades in glioma. (**C**) The expression of 46 PRLs between molecular subtypes. (**D**) The expression of 46 PRLs between normal tissues and glioma tissues. ^*^p < 0.05, ^**^p < 0.01, ^***^p < 0.001.

**Figure 2:**
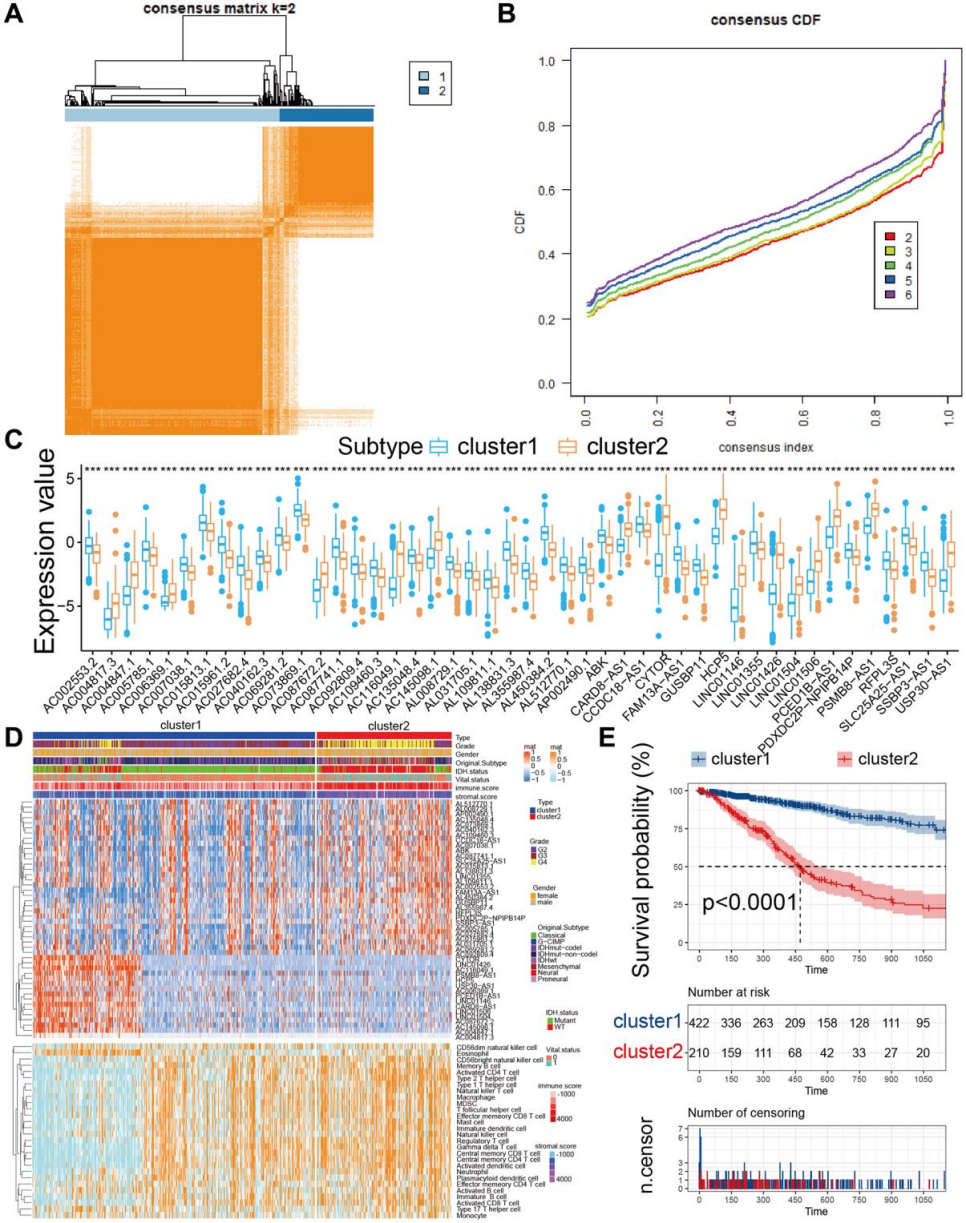
**Subgroups of glioma related by PRLs.** (**A**) The consensus score matrix of all samples in TCGA cohorts at k = 2. (**B**) Consensus clustering cumulative distribution function (CDF) for k = 2–6 in TCGA cohort. (**C**) The expression of 46 PRLs between two cluster groups. (**D**) The heatmap for 46 PRLs and 22 kinds of immune cells. (**E**) KM curves for the two cluster groups (Log-rank test, *p* = 0.0001).

Graphical abstract showed the overview of this study. We evaluated 14391 lncRNAs by performing the RNA-seq of the glioma patient samples from the Cancer Genome Atlas (TCGA). We also took 11 pyroptosis related genes from the previous literatures. Then we identified 46 PRLs by carrying out Pearson correlation between the lncRNAs and the PRLs applying |R|>0.7 and *P* < 0.05 as the selective standards (Figure 1A, Supplementary Table 1). These were *AC002553.2*, *AC004817.3*, *AC004847.1*, *AC005785.1*, *AC006369.1*, *AC007038.1*, *AC015813.1*, *AC015961.2*, *AC027682.4*, *AC040162.3*, *AC069281.2*, *AC073869.1*, *AC087672.2*, *AC087741.1*, *AC092809.4*, *AC109460.3*, *AC116049.1*, *AC135048.4*, *AC145098.1*, *AL008729.1*, *AL031705.1*, *AL109811.1*, *AL138831.3*, *AL355987.4*, *AL450384.2*, *AL512770.1*, *AP002490.1*, *ARHGAP27P1-BPTFP1-KPNA2P3* (ABK), *CARD8-AS1*, *CCDC18-AS1*, *CYTOR*, *FAM13A-AS1*, *GUSBP11*, *HCP5*, *LINC01146*, *LINC01355*, *LINC01426*, *LINC01504*, *LINC01506*, *PCED1B-AS1*, *PDXDC2P-NPIPB14P*, *PSMB8-AS1*, *RFPL3S*, *SLC25A25-AS1*, *SSBP3-AS1*, *USP30-AS1*. Moreover, we examined the correlation between the expression patterns of these regulators and molecular features. All PRLs were clearly distinguished in tumor grading (Figure 1B). Of the 46 PRLs, 44 revealed significant differences in groups of molecular subtypes, while *AC005785.1*, and *PDXDC2P-NPIPB14P* did not (Figure 1C). Of the 46 PRLs, 38 showed significant differences between tumor and normal tissues, while *AC006369.1*, *AC007038.1*, *AC116049.1*, *AL008729.1*, *AL031705.1*, *AL138831.3*, *LINC01506* and *SSBP3-AS1* did not (Figure 1D). These findings also correspond to the poor prognosis of cluster2 as shown in Figure 2E. Taken together, these results suggested that cross-talk among the PRLs plays an important role in the development of glioma.

### Identification of PRLs pattern in glioma

**Figure 3:**
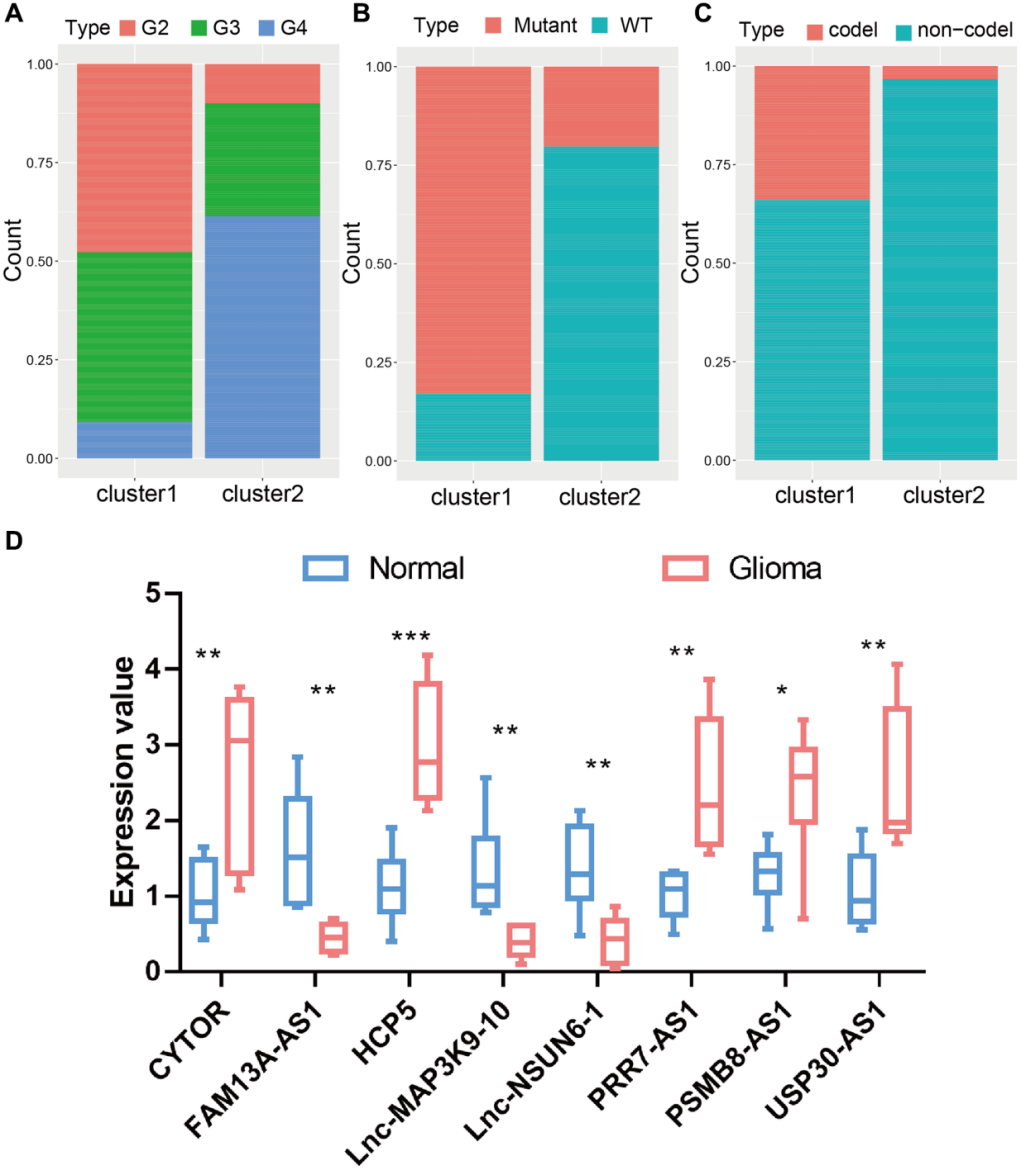
**The proportion of patients with grades, IDH1 mutation type, and 1p19q codel status in the high or low-risk groups in the TCGA cohort.** (**A**) The proportion of patients with grades mutation type in the high or low-risk groups. (**B**) The proportion of patients with IDH1 mutation type in the high or low-risk groups. (**C**) The proportion of patients with 1p19q codel status in the high or low-risk groups. (**D**) The expression value of 8 lncRNA in glioma tissues and normal brain tissues.

To conduct further studies on the expression features of PRLs in glioma, we classified glioma patients using a consensus clustering algorithm the expression profiles of 46 PRLs. We chose k = 2 for stable clustering of PRLs according to their cumulative distribution functions. Subsequently, we applied two modulation patterns by using the unsupervised clustering method, containing 484 cases in PRLs cluster 1 and 214 cases in PRLs cluster 2 (Figure 2A, 2B). Cluster 1 has a higher survival advantage than cluster 2 (Figure 2E). We further proceeded heatmap and quantitative analysis of the expression values of the 46 PRLs. Among the 46 PRLs, 14 PRLs in cluster 2 were significantly higher than in cluster 1, and the remaining 32 PRLs which were the reverse (Figure 2C, 2D, Supplementary Table 2). Then, we calculated the distribution of grades, IDH and 1p19q in the two clustering groups respectively, and found that G4, IDHwt, and non-codel accounted for a high proportion of cluster 2 (Figure 3A–3C). The strong relationship between our PRLs subgroups and clinical features further demonstrates the precision and robustness of our identification of PRLs patterns in glioma.

### The immune landscape of PRLs groups

The interplay between PRLs groups and the immune system relies on a complicated cellular cross-talk involving PRLs and immune cells [14]. Therefore, we assessed the immune status of gliomas by ssGSEA base on the infiltration of immune cells in the tumor organization, and classified glioma samples into two subgroups by consensual clustering. The results showed that we differentiated immune cell infiltration between the two cluster groups and there was significant enrichment of immune cell infiltration observed in cluster2 group, the risk score gradually increased with tumor grades (Figure 2D, Supplementary Figures 1B and 3B). Immune checkpoints (ICPs) are crucial for cancer immunotherapy, and a number of ICPs agonists and antagonists being evaluated in clinical oncology [15–17], we further performed their expression profile in distinct subtypes. Except for *TNFRSF25*, *TNFSF18*, *TNFSF9*, and *VSIR*, all the ICPs were overexpressed in the TCGA cohort high immunity group (Supplementary Figure 1A). Thus, patients in cluster2 may have a stronger immune response to tumorigenesis and tumor progression and therefore may benefit more from immune checkpoint inhibitors therapy than patients in cluster1. Then, we quantitatively analyzed the ESTIMATEscores of the two clusters and found that the Immunescore and Stromalscore of cluster 2 were significantly higher than that of cluster 1, and the tumor purity was just the contrary (Supplementary Figure 1C). The above results confirm the high immune status of cluster2 and the low immune status of cluster1.

### The mutation landscape of PRLs groups

**Figure 4:**
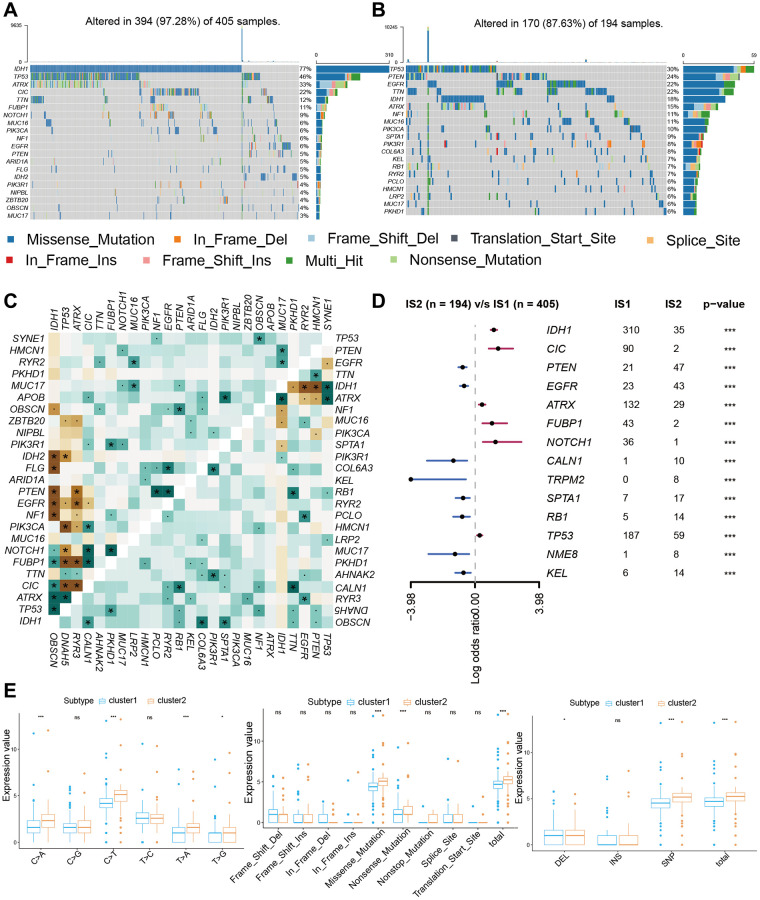
**The mutation landscape of two cluster groups.** (**A**, **B**) Waterfall diagram displays the mutation landscape of the top 20 most commonly mutation genes. (**C**) The heatmap analyzes the mutual co-occurrence and exclusion mutations of the top 25 commonly mutation genes. (**D**) The forest plot shows the top 10 most distinctively mutation genes between the two groups. (**E**) Boxplots displaying the comparisons of mutation frequencies of each mutation type classified by effects, SNV, DEL, INS, and SNP.

**Figure 5:**
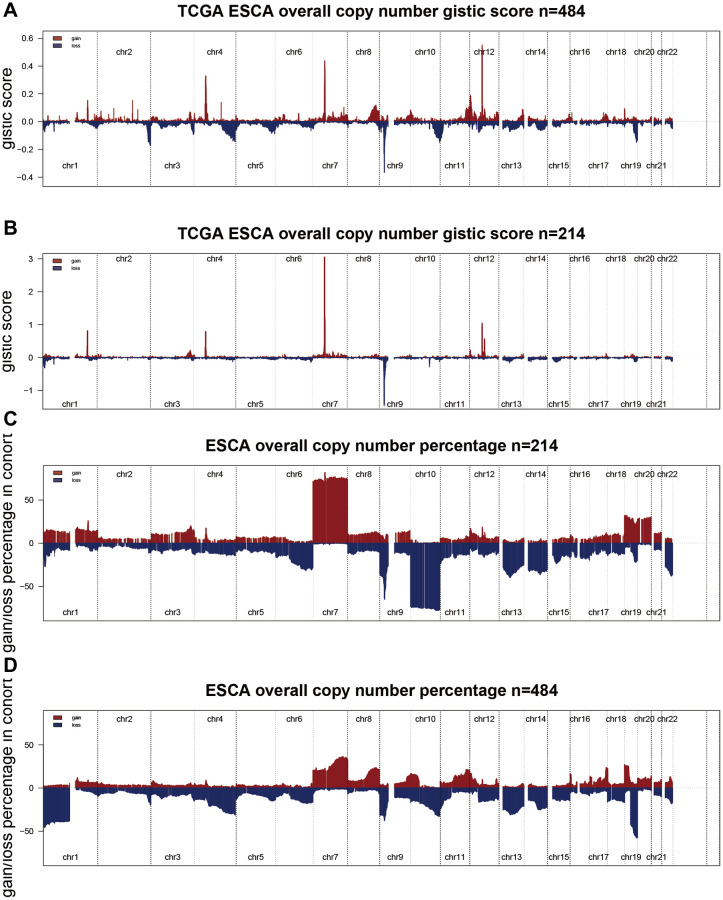
**Comprehensive analyses of copy number variation between two cluster groups.** (**A**, **B**) Detection and comparison of the percentage of significant scores between the two groups. (**C**, **D**) Detection and comparison of the percentage of significant gains and losses between the two groups.

Related papers indicate that immunity state may also contact with mutation [18]. Greater tumor mutational burden (TMB) and somatic mutation rates are linked to stronger cancer immunity [19]. We performed variation rate analysis in both groups in order to investigate whether immune infiltration status was associated with mutation rate. Among the two subtypes, cluster1 had the higher mutation rate (97.28%) than that in cluster2 (87.63%) (Figure 4A, 4B). The IDH1 mutation was higher in cluster1 (77%) than cluster2 (18%), IDH1 mutation dramatically indicated the outcome of glioma patients, so the distinction in IDH1 mutation between two cluster subtypes may be one of the reasons that contribute to the survival of patients [20]. This finding is in agreement with the results of the survival analysis of the two clusters in Figure 2E. In addition, we explored the landscape of co-occurrence using the top first 25 mutation genes with the comet algorithm. Twelve pairs of cases (*IDH1*-*IDH2*, *IDH1*-*FLG*, *IDH1*-*PTEN*, *IDH1*-*EGFR*, *IDH1*-*NF1*, *PIK3CA*-*TP53*, *FUBP1*-*TP53*, *FUBP1*-*ATRX*, *CIC*-*TP53*, *CIC*-*ATRX*, *IDH1*-*EGFR*, *IDH1*-*PTEN*) compared with prevalent mutually exclusive mutations showed mutually exclusive mutations, indicating that they may have superfluous impact in the common pathway and a selected advantage of retaining the mutation copy between them (Figure 4C). After checking transcriptional changes in the two subgroups mentioned above, the presence of genomic level distinction between the two subgroups was further investigated. Somatic mutations, comprising single nucleotide polymorphisms (SNP), single nucleotide variants, insertions, and deletions, were computed and shown applying the “maftools” algorithm [21]. The SNPs and Total in cluster2 group were also exceeded by those in cluster1 group, while the major portion of genomic variants were missense mutations (60%) in the two subgroups. As for SNVs, all samples were examined, and C>T was the prevalent type in both groups. For most of the types of SNV (C>A, C>T, T>A, T>G), the mutation number was clearly higher in the cluster2 group than in cluster1 group (Figure 4E). Moreover, the samples in the cluster1 subgroup have a remarkably higher level of variant allele fractions (VAFs) than those in the cluster2 subgroup (Supplementary Figure 2B), which had been thought to be linked to cancer progression and worse results, supporting the discovery of relatively superior tumor purity and inferior heterogeneity in the cluster1 samples. This finding echoes the answer we got in Figure 4C. The proportion between transversion and transition in all SNVs were roughly 3:1 and maintained stabilized in both subgroups (Supplementary Figure 2A). More interesting is that several genes had distinct mutation rates between the two subgroups. In terms of outcomes, the top 10 genes were shown in Figure 4D. Furthermore, IDH1 is another classic example demonstrating the distinct mutation sites between two subgroups (Supplementary Figure 2C) and the plausible chain reaction of the variance in prognostic effect. Eventually, we evaluated driver gene for the two subtypes, and the findings indicated that the dominant driver genes of cluster1 subgroup were *IDH1*, *IDH2*, and *KRT15*, meanwhile, the driver gene of cluster2 subgroup was IDH1 (Supplementary Figure 2D). With removing germline CNV, remarkable gains and losses were observed for each cohort with a threshold FDR < 0.05. Through comparison, we detected more regional alterations in the cluster1 subgroup (Figure 5A, 5B, Supplementary Table 3). By computing the frequency of each CNV in all patients, we discovered that 7q gain and 10p loss were the most common CNVs in cluster1 subgroup; whereas 1p loss, 7q gain and 19p loss were also among the most common changes occurred in cluster2 subgroup (Figure 5C, 5D, Supplementary Table 4). We found significant differences between two subgroups in most mutation features, further demonstrating the accuracy of grouping by PRLs in predicting mutational features.

### Identification hub PRLs in glioma and construction of pyroptosis related LncRNA prognostic model

**Figure 6:**
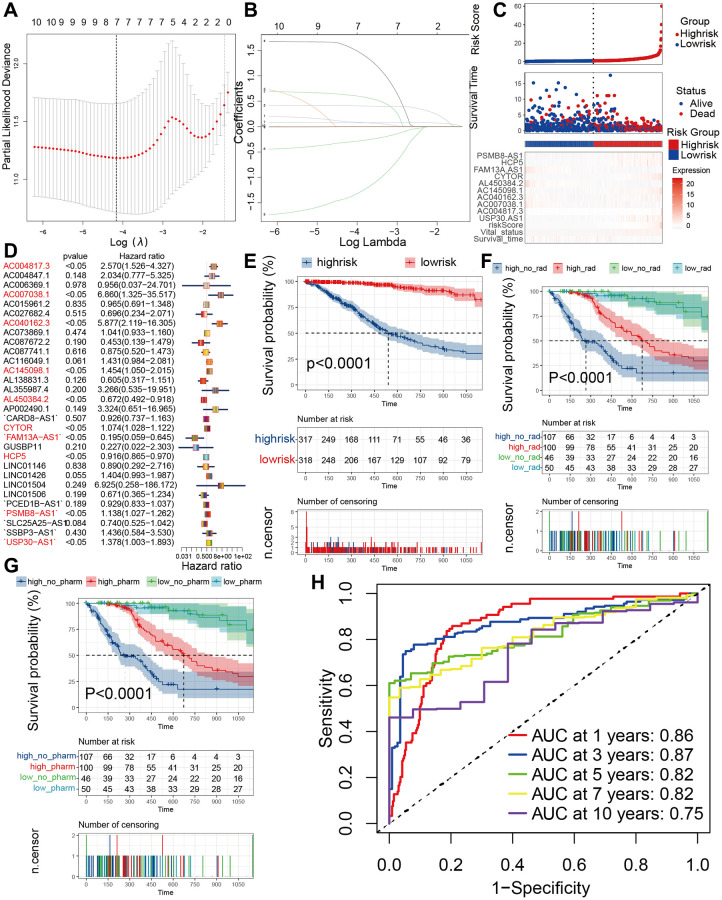
**Predicting patient prognosis in the TCGA cohort based on PRLPM.** (**A**) Regression coefficient profiles of identified pyroptosis immune regulators in the TCGA cohort. (**B**) Ten-time cross-validation for tuning parameter selection in the TCGA cohort. (**C**) Patients were divided into high and low-risk subgroups according to median level of PRLPM riskscore in train set; heatmap of 9 PRLs. (**D**) multivariate cox regression analyses of the association between PRLs and OS of patients in the TCGA cohort. (**E**) KM curve plot of OS for patients in high and low-risk subgroups. (**F**, **G**) Survival analyses for subgroup patients stratified by both PRLPM riskscore and treatment with radiotherapy (**F**) and pharmacological chemotherapy (**G**) in the TCGA cohort. (**H**) The timeROC curve to evaluate the prognostic value of PRLPM riskscore in TCGA cohort.

To further obtain more accurate PRLs, we conducted univariate and multi-variate cox regressions from 46 PRLs and yielded 10 hub PRLs (Figure 6D). The 10 hub PRLs were *AC004817.3* (*lnc-MAP3K9-10*), *AC007038.1*, *AC040162.3*, *AC145098.1* (*PRR7-AS1*), *AL450384.2* (*NSUN6*), *CYTOR*, *FAM13A-AS1*, *HCP5*, *PSMB8-AS1*, *USP30-AS1*. To verify the reliability of these 10 hub PRLs for glioma patients, we validated these 10 PRLs in human glioma samples. *AC007038.1* and *AC040162.3* were not found with corresponding sequences due to their relatively short discovery time, and we performed PCR analysis on the remaining 8 lncRNAs, which showed consistent expression with the TCGA database (Figure 3D). Seeing that this has the stability of lncRNA expression, it lays the foundation for our further analysis. We constructed pyroptosis related lncRNA prognostic model (PRLPM) by lasso regression for 10 hub PRLs (Figure 6A, 6B). Moreover, we built the riskscore system according to PRLPM to classify patients into high and low risk groups based on PRLPM (Supplementary Table 5). Kaplan Meier survival analyses revealed that overall survival (OS) was worse in the high-risk cohort in comparison to the low-risk cohort (Figure 6E), the same result was obtained in Chinese Glioma Genome Atlas (CGGA) cohort (Supplementary Figure 3F). The survival preference in the low-risk group was superior to the high-risk group, whatever treatment was used (Figure 6F, 6G). Meanwhile, the timeROC showed the mean AUC values of prognosis predictions on TCGA cohort reached 0.86, 0.87, 0.82, 0.82 and 0.75 (Figure 6H), the AUC values on CGGA were 0.7, 0.7, 0.69, 0.71, and 0.79 (Supplementary Figure 3E). Distribution of PRLPM riskscore and OS also verified the survival analyses, the findings suggested that the expression of 10 hub PRLs was remarkably positively associated with PRLPM riskscore and OS (Figure 6C). From the above results, we found that PRPLPM has high accuracy in predicting the risk profile and prognosis of glioma patients.

### The relationship between riskscore and clinical traits

We ordered the samples on the basis of their riskscore (rank low to high riskscore) and examined whether any demographic/molecular/clinical characteristics were associated with PRLPM in two public cohorts (Supplementary Figures 4A and 3A, Supplementary Table 6). We further quantitatively analyzed the relevance between risk scores and molecular/clinical traits. In the TCGA cohort, the results revealed that the PRLPM riskscore gradually increased with increasing grade, with lower PRLPM riskscore in patients with IDH1mut than that in IDH1wt patients and significantly higher PRLPM riskscore in 1p19q non-codel patients than that in 1p19q codel patients (Supplementary Figure 4B–4D). In the CGGA, the findings revealed that the PRLPM riskscore gradually increased with increasing grade, with lower PRLPM riskscore in patients with IDH1mut than that in IDH1wt patients and significantly higher PRLPM riskscore in recurrent and secondary patients than that in primary patients (Supplementary Figure 3B–3D). The proportion of tumor grading within the two subgroups showed that G4 and G3 were only found in the high-risk group. The proportion of IDH1mut in both subgroups revealed that IDH1mut was in predominant in the low-risk group. The rate of 1p19q codel was considerably higher in the low-risk than in the high-risk group (Supplementary Figure 4E–4G). These results demonstrate that PRLPM riskscores can respond to the demographic/molecular/clinical feature of glioma.

### The relationship between riskscore and tumor stemness indices

Stems differed between PRLPM groups; specifically, the dedifferentiated phenotype was significant in the high-risk group, while the differentiated phenotype was significant in the low risk group (Supplementary Figure 5A, 5B). Then, we calculated the TMB and found that the TMB in the high-risk group was obviously higher than that in the low-risk group. Moreover, mRNAsi, EREG-mDNAsi, mDNAsi, EREG-mRNAsi, ENHsi, TMB, and DMPsi were actively and significantly related to the PRLPM riskscore, and the relevance were significant at 0.45, 0.49, 0.5, 0.018, 0.5, 0.45, 0.39, respectively (Supplementary Figures 5C–5H, 6C, Supplementary Table 7). Considering that the PRLs characterized an immune activation phenotype of glioma, we applied the algorithms to evaluate the effectiveness of the PRLs signatures in forecasting ICB responsiveness in glioma and used SubMap to compare the prediction results [22, 23]. Consequently, our PRLs had comparable performance in predicting the glioma response to anti-PD1 and anti-CTLA4 treatments (Supplementary Figure 6D), indicating that stratification based on PRLs has the possible to recognize ICB reactors. In conclusion, there were distinct differences in the extent of tumor differentiation, and TMB, between the PRLPM groups.

### Comparison of differentially expressed genes and sensitivity to chemotherapeutic agents in high and low-risk groups

We examined the differential analysis for the both groups. Enrichment analysis was performed to forecast the potential functions of differentially expressed genes (DEGs) between high and low-risk groups, and as expected, the DEGs were concentrated in carcinogenic and immune pathways, for example neutrophil degranulation, neutrophil activation involved in immune response, wnt signaling pathway, p53 signaling pathway, Apoptosis, HALLMARK_APOPTOSIS, PI3K_AKT_MTOR_SIGNALING, HALLMARK_COMPLEMENT (Supplementary Figure 7A–7D).

Different PRLPM subgroups should guide clinical therapy. Therefore, we made a comparison of the sensitivity of 30 anticancer drugs between the both groups to identify potential glioma treatment approaches. A total of 22 chemotherapy drugs had remarkably distinct IC50 estimates between two groups (Supplementary Figure 6A). Patients in the low-risk group may be sensitive to these drugs. In this context, these agents have the potential to be used in the future for the treatment of low-risk PRLPM. Moreover, we find that the TMB in high-risk group was obviously higher than low-risk group (Supplementary Figure 6B). This result indicated high risk had better therapeutic effect than low risk in immunotherapy.

### The efficacy of the PRLPM riskcore across tumor types

**Figure 7:**
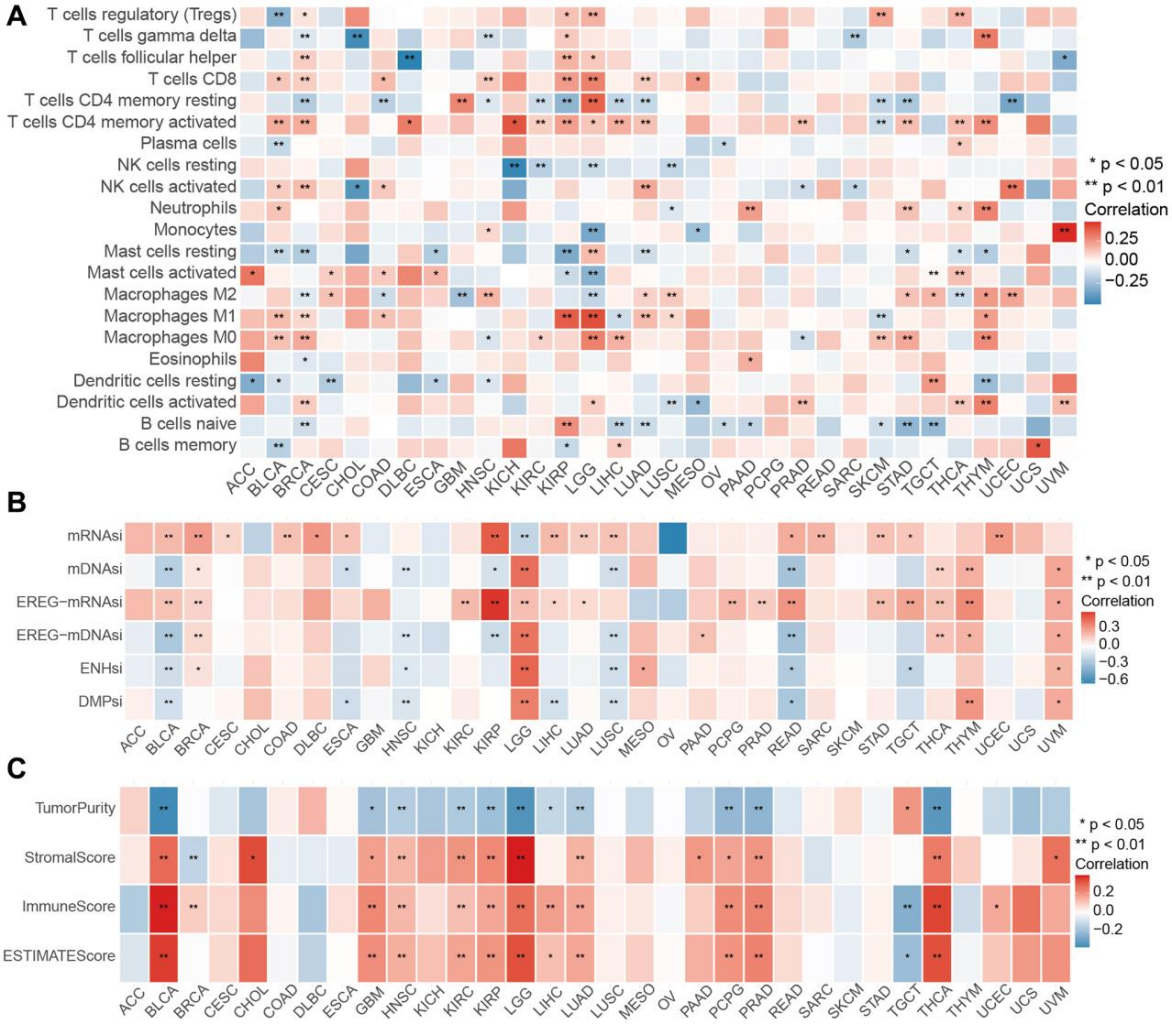
**Performance of PIPM riskscore across tumor types.** (**A**) Association between the PIPM riskscore and immune cells for each cancer type. (**B**) Association between the PIPM riskscore and stemness indices for each cancer type. (**C**) Correlations between the PIPM riskscore and ESTIMATEscore for each cancer type.

Considering the strong correlation between PRLPM and immunity described above, we further examined the validity of the PRLPM scoring system in different tumor types. The percentage of 22 immune cells was characterized as immune infiltration. We computed the relevance between the 22 immune cells percentage and the PRLPM riskcores and revealed the distinct trends in the relevance for pan-cancer, except PCPG and READ. The percentage of T cells CD8, T cells CD4 memory active, T cells CD4 memory resting, Macrophages M1, B cells naive were relevant to the PRLPM riskcores of most cancer types. Interestingly, these cells belonged to the antitumor type, to some extent, suggesting that the PRLs promote tumor immunity (Figure 7A). Furthermore, we identified a correlation between PRLPM riskscores and stem cell indices for all tumors except for ACC, KICH, SKCM, and UCS (Figure 7B). We found the significant relationship between the ESTIMATEscore and the PRLPM riskcores in addition to PCPG, and READ (Figure 7C). Such cancers with high mDNAsi were expected to be hypersensitive to immune checkpoint treatment. We thoroughly assessed the correlation of the PRLPM scoring system and the pan-cancer, and deeply explored the outstanding features of the PRLPM riskscore in pan-cancer, and laid the basis for the wide utilization of PRLPM riskscore in tumor.

## DISCUSSION

In our study, we first identified 10 PRLs that notably associated with OS based on the univariate cox regression of the expression of PRLs in the glioma samples from TCGA. Pyroptosis may be the panacea for the therapy of many cancers in the future [4]. The induction of pyroptosis could notably inhibit cancer proliferation and patient aggravation, even in chemoresistant cases, since apoptosis rather than pyroptosis is the most frequent mechanism of chemotherapeutic drugs insensitivity. Nevertheless, the regulatory mechanism of pyroptosis remains largely unclear, especially in the area of non-coding RNAs, particularly lncRNAs. Jirong Wang et al. revealed that the *AL450384.2* not only is the immune-related lncRNA and can be used as a prognostic marker for bladder cancer [24]. Brian J Reon et al. found *CYTOR* to be a potential biomarker and therapeutic target in glioblastoma and other tumor types, combining its prognostic potential and ability to promote invasion [25]. This group recently reported that *FAM13A-AS1* also was an autophagy-related lncRNA and accurately predicted the prognosis of BCLA patients [26]. *HCP5* facilitates cell proliferation and restrains apoptosis via *miR-27a-3p*/*IGF-1* axis in human granulosa-like tumor cell line *KGN* [27]. *PSMB8-AS1* is involved in pancreatic cancer progression through modulating *miR-382-3p*/*STAT1*/*PD-L1* axis [28]. *USP30-AS1* is involved in mitochondrial quality control in glioblastoma cells [29]. All these lncRNAs were significantly associated with tumor proliferation, and FAM13A-AS1 was not the only PRLs but also autophagy-related lncRNAs, further indicating the important role of our screened lncRNAs in the development of glioma. Hence, systematic selection for potential PRLs is imperative to accelerate the development of this field.

The molecular mechanism of pyroptosis has triggered a strong inflammatory response and was considered as an ICD form in some cases and several studies established clinical prognostic models that use transcriptomic expression levels of pyroptosis regulators to predict survival outcomes of cancer patients. Nevertheless, we were the first to identify novel PRLs and construct a clinical prognostic model in glioma. We built prognostic models according to the expression profile of the 10 PRLs. The AUCs of the PRLPM ranged from 0.75 to 0.87 and consistent with the 1- to 10-year follow-up, which is hopeful for clinical translation.

Crosstalk between pyroptosis and immune microenvironment remodeling has been shown in previous studies [30]. It is noteworthy that we also uncovered a relevance between two cluster groups and the immune microenvironment and mutation landscape, which may instruct distinct therapeutic approaches in the both groups. For instance, more NK cells and CD8+ infiltrated the cluster2 group, but CTLA4 expression was also increased in this cluster group and may inhibit the antitumor capabilities of these cytotoxic cells. The PRLPM gained by the subgraph algorithm was better in predicting immune checkpoint treatment. Hence, immune checkpoint inhibitors that target CTLA4, such as tremelimumab and ipilimumab, may be the best therapeutic approach for these patients. We observed that there are several chemotherapy agents with better sensitivity to high and low-risk groups, respectively, so that appropriate chemotherapy agents can be chosen based on distinct risk groups. The PRLPM riskscore system, which reflects individual pyroptosis modification patterns, reliably stratifies the stemness regulatory patterns with survival differences, as well as susceptibility to immunotherapy and radio-chemotherapy.

The performance of this scoring strategy applied to other cancer types also showed survival advantage of PRLPM riskscore and the correlation between PRLPM riskscores and stemness and immunity. In a sense, the PRLPM riskscore also reflects the stemness and immunity of cancers. The relevance between immune cells, stemness and PRLPM riskscore may suggest that both phenotypes are influenced by the PRLs in most tumors, leading to an uncontrolled immune disease and dedifferentiation defined by loss of structure of origin [31].

The current study has several strengths. First, we are the first team to construct an PRLs prognosis model in glioma. This model provided high diagnostic accuracy and can be applied for clinical translation. Second, the PRLPM may also guide clinicians to identify appropriate drugs for the therapy of glioma by comparing the sensitivity of patients in high and low-risk populations to 30 common anti-cancer drugs. Nevertheless, the current algorithm is mainly based on bioinformatics analysis and further preclinical studies are needed to validate.

Certainly, the current research has its limitations. Many previous bioinformatic literatures also examined lncRNA-based features in an individual TCGA and obtained considerable attention [32–34]. We selected PRLs according to the coefficient of coexpression relevance. Nevertheless, a more straightforward selection strategy is to compare the pool of altered lncRNAs in glioma samples with and without pyroptosis induction. Unfortunately, there are no clinically approved targeted agents that specifically cause pyroptosis, which hampers the viability of this selection strategy.

## CONCLUSIONS

In summary, the current research was the first to recognize the PRLs pool and build an PRLPM that displayed high diagnostic precision in forecasting OS in glioma patients. It is anticipated that future studies will explore how lncRNA regulates pyroptosis and the potential regulatory mechanisms that influence the efficacy of focal death inducer therapy. We hope that the usefulness of PRLPM can also be confirmed by other clinical studies in the future.

## METHODS

### Data extraction

The RNA_seq and clinical data of 698 glioma samples were obtained from TCGA, clinical data of 1018 glioma samples were downloaded from CGGA. Mutation rate and CNV frequency were gathered from cBioPortal. Clinical data of glioma patients, including age, sex, grades, OS and survival status was also collected for subsequent analyses.

### Recognizing pyroptosis regulators

Access to relevant literature, we collected 11 genes (*CASP1*, *CASP3*, *CASP4*, *CASP5*, *CASP8*, *GSDMB*, *GSDMC*, *GSDMD*, *GSDME*, *GZMA*, *GZMB*), which are closely related to pyroptosis and serves as pyroptosis genes [35–37].

### Consensus clustering

Consensual clustering utilizes the k-means technique to determine specific pyroptosis patterns associated with the expression of pyroptosis regulators. Both stability and number were decided by the consensus clustering algorithm applying the “ConsensusClusterPlus” package [38].

### Immune infiltration

The relative infiltration of 28 immune cell types in TME was described using ssGSEA. Signature gene panels were obtained for each immune cell type from a recent paper [39].

### Analyses of mutation subtypes

Somatic mutation and CNV profiles were downloaded from the TCGA data portal. Analysis of somatic mutation data sorted by mutation annotation format was performed using the R package “maftools” [40, 41].

### PRLs prognostic model predicts effective response to chemoradiotherapy, the analyses of relationship between clinical characteristics, tumor stemness indices, tumor mutation burden, and prognostic model riskscore

The prognostic model was developed by lasso regression for PRLs, and we analyzed the survival, ROC, risk factor, stemness, the response to chemoradiotherapy. The Kruskal-Wallis test was used to evaluate the difference between TMB, tumor stemness index and the prognostic model generated by lasso algorithm, and Pearson correlation was used to evaluate the correlation between tumor stemness index, TMB and prognostic model riskscore. Furthermore, the dedication to OS were computed by Kaplan-Meier algorithm. We also continued to analyze the association of prognostic model riskscores with clinical characteristics separately.

### Significance of the pyroptosis related lncRNA prognostic model in drug sensitivity

To estimate PRLPM in the clinical treatment of glioma, we used the method developed by Geeleher et al. [42] and the corresponding R package ‘prophetic’ to compute TCGA items for the IC50 of widely applied chemotherapy agents in the glioma dataset [43, 44]. The AJCC guidance suggested 30 common anti-tumor medications, like Cisplatin, Vinblastine, Imatinib, Adriamycin and/or cancer therapy. Wilcoxon signed rank method was employed to compare the variation in IC50 values of common antineoplastic agents between PRLPM high- and low-risk groups, and the results are presented as box plots.

### Real-time quantitative PCR

The RNA of 6 human glioma tissues and corresponding peritumoral tissues (Normal tissue) were reverse-transcribed by RT reagent Kit gDNA Eraser (TaKaRa), and SYBR-Green (TaKaRa) detection was performed. The PCR primers were shown in Supplementary Table 8.

### Statistical analysis

All data were performed with R version 4.0.5 and its corresponding packages. ESTIMATEscore was computed by using the ‘estimate’ package [45]. The lasso cox regression was carried out using the ‘glmnet’ package [46]. The data were analyzed using proper statistical criterion. Multiple-test adjustment was applied utilizing the FDR method.

### Availability of data and materials

We organized the original data and uploaded it to Github. This is the URL we uploaded: https://github.com/shuaima1991/pyroptosis-related-lncRNA.git.

## Supplementary Materials

Supplementary Figures

Supplementary Tables

## References

[1] Han B, Meng X, Wu P, Li Z, Li S, Zhang Y, Zha C, Ye Q, Jiang C, Cai J, Jiang T. ATRX/EZH2 complex epigenetically regulates FADD/PARP1 axis, contributing to TMZ resistance in glioma. Theranostics. 2020; 10:3351–65. 10.7150/thno.41219 32194873 PMC7053195

[2] Ma S, Ba Y, Ji H, Wang F, Du J, Hu S. Recognition of Tumor-Associated Antigens and Immune Subtypes in Glioma for mRNA Vaccine Development. Front Immunol. 2021; 12:738435. 10.3389/fimmu.2021.738435 34603319 PMC8484904

[3] Fan JX, Deng RH, Wang H, Liu XH, Wang XN, Qin R, Jin X, Lei TR, Zheng D, Zhou PH, Sun Y, Zhang XZ. Epigenetics-Based Tumor Cells Pyroptosis for Enhancing the Immunological Effect of Chemotherapeutic Nanocarriers. Nano Lett. 2019; 19:8049–58. 10.1021/acs.nanolett.9b03245 31558023

[4] Loveless R, Bloomquist R, Teng Y. Pyroptosis at the forefront of anticancer immunity. J Exp Clin Cancer Res. 2021; 40:264. 10.1186/s13046-021-02065-8 34429144 PMC8383365

[5] Shao W, Yang Z, Fu Y, Zheng L, Liu F, Chai L, Jia J. The Pyroptosis-Related Signature Predicts Prognosis and Indicates Immune Microenvironment Infiltration in Gastric Cancer. Front Cell Dev Biol. 2021; 9:676485. 10.3389/fcell.2021.676485 34179006 PMC8226259

[6] Wan P, Su W, Zhang Y, Li Z, Deng C, Li J, Jiang N, Huang S, Long E, Zhuo Y. LncRNA H19 initiates microglial pyroptosis and neuronal death in retinal ischemia/reperfusion injury. Cell Death Differ. 2020; 27:176–91. 10.1038/s41418-019-0351-4 31127201 PMC7206022

[7] Zhang Y, Liu X, Bai X, Lin Y, Li Z, Fu J, Li M, Zhao T, Yang H, Xu R, Li J, Ju J, Cai B, et al. Melatonin prevents endothelial cell pyroptosis via regulation of long noncoding RNA MEG3/miR-223/NLRP3 axis. J Pineal Res. 2018; 64:e12449. 10.1111/jpi.12449 29024030

[8] Kopp F, Mendell JT. Functional Classification and Experimental Dissection of Long Noncoding RNAs. Cell. 2018; 172:393–407. 10.1016/j.cell.2018.01.011 29373828 PMC5978744

[9] Peng Z, Liu C, Wu M. New insights into long noncoding RNAs and their roles in glioma. Mol Cancer. 2018; 17:61. 10.1186/s12943-018-0812-2 29458374 PMC5817731

[10] Li Z, Zhang J, Zheng H, Li C, Xiong J, Wang W, Bao H, Jin H, Liang P. Modulating lncRNA SNHG15/CDK6/miR-627 circuit by palbociclib, overcomes temozolomide resistance and reduces M2-polarization of glioma associated microglia in glioblastoma multiforme. J Exp Clin Cancer Res. 2019; 38:380. 10.1186/s13046-019-1371-0 31462285 PMC6714301

[11] Voce DJ, Bernal GM, Wu L, Crawley CD, Zhang W, Mansour NM, Cahill KE, Szymura SJ, Uppal A, Raleigh DR, Spretz R, Nunez L, Larsen G, et al. Temozolomide Treatment Induces lncRNA MALAT1 in an NF-κB and p53 Codependent Manner in Glioblastoma. Cancer Res. 2019; 79:2536–48. 10.1158/0008-5472.CAN-18-2170 30940658 PMC6522287

[12] Zhang Q, Huang XM, Liao JX, Dong YK, Zhu JL, He CC, Huang J, Tang YW, Wu D, Tian JY. LncRNA HOTAIR Promotes Neuronal Damage Through Facilitating NLRP3 Mediated-Pyroptosis Activation in Parkinson's Disease via Regulation of miR-326/ELAVL1 Axis. Cell Mol Neurobiol. 2021; 41:1773–86. 10.1007/s10571-020-00946-8 32968928 PMC11444004

[13] Mao Q, Liang XL, Zhang CL, Pang YH, Lu YX. LncRNA KLF3-AS1 in human mesenchymal stem cell-derived exosomes ameliorates pyroptosis of cardiomyocytes and myocardial infarction through miR-138-5p/Sirt1 axis. Stem Cell Res Ther. 2019; 10:393. 10.1186/s13287-019-1522-4 31847890 PMC6918658

[14] Legrand AJ, Konstantinou M, Goode EF, Meier P. The Diversification of Cell Death and Immunity: Memento Mori. Mol Cell. 2019; 76:232–42. 10.1016/j.molcel.2019.09.006 31586546

[15] Galluzzi L, Vitale I, Warren S, Adjemian S, Agostinis P, Martinez AB, Chan TA, Coukos G, Demaria S, Deutsch E, Draganov D, Edelson RL, Formenti SC, et al. Consensus guidelines for the definition, detection and interpretation of immunogenic cell death. J Immunother Cancer. 2020; 8:e000337. 10.1136/jitc-2019-000337 32209603 PMC7064135

[16] Hodges TR, Ott M, Xiu J, Gatalica Z, Swensen J, Zhou S, Huse JT, de Groot J, Li S, Overwijk WW, Spetzler D, Heimberger AB. Mutational burden, immune checkpoint expression, and mismatch repair in glioma: implications for immune checkpoint immunotherapy. Neuro Oncol. 2017; 19:1047–57. 10.1093/neuonc/nox026 28371827 PMC5570198

[17] Thorsson V, Gibbs DL, Brown SD, Wolf D, Bortone DS, Ou Yang TH, Porta-Pardo E, Gao GF, Plaisier CL, Eddy JA, Ziv E, Culhane AC, Paull EO, et al, and Cancer Genome Atlas Research Network. The Immune Landscape of Cancer. Immunity. 2018; 48:812–30.e14. 10.1016/j.immuni.2018.03.023 29628290 PMC5982584

[18] Huang X, Zhang G, Tang T, Liang T. Identification of tumor antigens and immune subtypes of pancreatic adenocarcinoma for mRNA vaccine development. Mol Cancer. 2021; 20:44. 10.1186/s12943-021-01310-0 33648511 PMC7917175

[19] Rooney MS, Shukla SA, Wu CJ, Getz G, Hacohen N. Molecular and genetic properties of tumors associated with local immune cytolytic activity. Cell. 2015; 160:48–61. 10.1016/j.cell.2014.12.033 25594174 PMC4856474

[20] Yan H, Parsons DW, Jin G, McLendon R, Rasheed BA, Yuan W, Kos I, Batinic-Haberle I, Jones S, Riggins GJ, Friedman H, Friedman A, Reardon D, et al. IDH1 and IDH2 mutations in gliomas. N Engl J Med. 2009; 360:765–73. 10.1056/NEJMoa0808710 19228619 PMC2820383

[21] Ma S, Wang F, Wang N, Jin J, Ba Y, Ji H, Du J, Hu S. Multiomics Data Analysis and Identification of Immune-Related Prognostic Signatures With Potential Implications in Prognosis and Immune Checkpoint Blockade Therapy of Glioblastoma. Front Neurol. 2022; 13:886913. 10.3389/fneur.2022.886913 35669882 PMC9165649

[22] Jiang P, Gu S, Pan D, Fu J, Sahu A, Hu X, Li Z, Traugh N, Bu X, Li B, Liu J, Freeman GJ, Brown MA, et al. Signatures of T cell dysfunction and exclusion predict cancer immunotherapy response. Nat Med. 2018; 24:1550–8. 10.1038/s41591-018-0136-1 30127393 PMC6487502

[23] Hoshida Y, Brunet JP, Tamayo P, Golub TR, Mesirov JP. Subclass mapping: identifying common subtypes in independent disease data sets. PLoS One. 2007; 2:e1195. 10.1371/journal.pone.0001195 18030330 PMC2065909

[24] Wang J, Shen C, Dong D, Zhong X, Wang Y, Yang X. Identification and verification of an immune-related lncRNA signature for predicting the prognosis of patients with bladder cancer. Int Immunopharmacol. 2021; 90:107146. 10.1016/j.intimp.2020.107146 33189610

[25] Reon BJ, Takao Real Karia B, Kiran M, Dutta A. *LINC00152* Promotes Invasion through a 3'-Hairpin Structure and Associates with Prognosis in Glioblastoma. Mol Cancer Res. 2018; 16:1470–82. 10.1158/1541-7786.MCR-18-0322 29991527 PMC6170721

[26] Sun Z, Jing C, Xiao C, Li T. An autophagy-related long non-coding RNA prognostic signature accurately predicts survival outcomes in bladder urothelial carcinoma patients. Aging (Albany NY). 2020; 12:15624–37. 10.18632/aging.103718 32805727 PMC7467376

[27] Chen Y, Zhang X, An Y, Liu B, Lu M. LncRNA HCP5 promotes cell proliferation and inhibits apoptosis via miR-27a-3p/IGF-1 axis in human granulosa-like tumor cell line KGN. Mol Cell Endocrinol. 2020; 503:110697. 10.1016/j.mce.2019.110697 31891769

[28] Zhang H, Zhu C, He Z, Chen S, Li L, Sun C. LncRNA PSMB8-AS1 contributes to pancreatic cancer progression via modulating miR-382-3p/STAT1/PD-L1 axis. J Exp Clin Cancer Res. 2020; 39:179. 10.1186/s13046-020-01687-8 32891166 PMC7487636

[29] Wang N, Li J, Xin Q, Xu N. USP30-AS1 contributes to mitochondrial quality control in glioblastoma cells. Biochem Biophys Res Commun. 2021; 581:31–7. 10.1016/j.bbrc.2021.10.006 34653676

[30] Wang Q, Wang Y, Ding J, Wang C, Zhou X, Gao W, Huang H, Shao F, Liu Z. A bioorthogonal system reveals antitumour immune function of pyroptosis. Nature. 2020; 579:421–6. 10.1038/s41586-020-2079-1 32188939

[31] Du J, Ji H, Ma S, Jin J, Mi S, Hou K, Dong J, Wang F, Zhang C, Li Y, Hu S. m6A regulator-mediated methylation modification patterns and characteristics of immunity and stemness in low-grade glioma. Brief Bioinform. 2021; 22:bbab013. 10.1093/bib/bbab013 33594424

[32] He A, He S, Peng D, Zhan Y, Li Y, Chen Z, Gong Y, Li X, Zhou L. Prognostic value of long non-coding RNA signatures in bladder cancer. Aging (Albany NY). 2019; 11:6237–51. 10.18632/aging.102185 31433789 PMC6738399

[33] Wei C, Liang Q, Li X, Li H, Liu Y, Huang X, Chen X, Guo Y, Li J. Bioinformatics profiling utilized a nine immune-related long noncoding RNA signature as a prognostic target for pancreatic cancer. J Cell Biochem. 2019; 120:14916–27. 10.1002/jcb.28754 31016791

[34] Zhang G, Fan E, Zhong Q, Feng G, Shuai Y, Wu M, Chen Q, Gou X. Identification and potential mechanisms of a 4-lncRNA signature that predicts prognosis in patients with laryngeal cancer. Hum Genomics. 2019; 13:36. 10.1186/s40246-019-0230-6 31416476 PMC6694645

[35] Shi J, Zhao Y, Wang K, Shi X, Wang Y, Huang H, Zhuang Y, Cai T, Wang F, Shao F. Cleavage of GSDMD by inflammatory caspases determines pyroptotic cell death. Nature. 2015; 526:660–5. 10.1038/nature15514 26375003

[36] Zhang Z, Zhang Y, Xia S, Kong Q, Li S, Liu X, Junqueira C, Meza-Sosa KF, Mok TMY, Ansara J, Sengupta S, Yao Y, Wu H, Lieberman J. Gasdermin E suppresses tumour growth by activating anti-tumour immunity. Nature. 2020; 579:415–20. 10.1038/s41586-020-2071-9 32188940 PMC7123794

[37] Zhou Z, He H, Wang K, Shi X, Wang Y, Su Y, Wang Y, Li D, Liu W, Zhang Y, Shen L, Han W, Shen L, et al. Granzyme A from cytotoxic lymphocytes cleaves GSDMB to trigger pyroptosis in target cells. Science. 2020; 368:eaaz7548. 10.1126/science.aaz7548 32299851

[38] Wilkerson MD, Hayes DN. ConsensusClusterPlus: a class discovery tool with confidence assessments and item tracking. Bioinformatics. 2010; 26:1572–3. 10.1093/bioinformatics/btq170 20427518 PMC2881355

[39] Jia Q, Wu W, Wang Y, Alexander PB, Sun C, Gong Z, Cheng JN, Sun H, Guan Y, Xia X, Yang L, Yi X, Wan YY, et al. Local mutational diversity drives intratumoral immune heterogeneity in non-small cell lung cancer. Nat Commun. 2018; 9:5361. 10.1038/s41467-018-07767-w 30560866 PMC6299138

[40] Koboldt DC, Zhang Q, Larson DE, Shen D, McLellan MD, Lin L, Miller CA, Mardis ER, Ding L, Wilson RK. VarScan 2: somatic mutation and copy number alteration discovery in cancer by exome sequencing. Genome Res. 2012; 22:568–76. 10.1101/gr.129684.111 22300766 PMC3290792

[41] Mayakonda A, Lin DC, Assenov Y, Plass C, Koeffler HP. Maftools: efficient and comprehensive analysis of somatic variants in cancer. Genome Res. 2018; 28:1747–56. 10.1101/gr.239244.118 30341162 PMC6211645

[42] Geeleher P, Cox NJ, Huang RS. Clinical drug response can be predicted using baseline gene expression levels and in vitro drug sensitivity in cell lines. Genome Biol. 2014; 15:R47. 10.1186/gb-2014-15-3-r47 24580837 PMC4054092

[43] Geeleher P, Cox N, Huang RS. pRRophetic: an R package for prediction of clinical chemotherapeutic response from tumor gene expression levels. PLoS One. 2014; 9:e107468. 10.1371/journal.pone.0107468 25229481 PMC4167990

[44] Ma S, Wang F, Wang N, Jin J, Yan X, Wang L, Zheng X, Hu S, Du J. Extended Application of Genomic Selection to Screen Multi-Omics Data for the Development of Novel Pyroptosis-Immune Signatures and Predicting Immunotherapy of Glioma. Front Pharmacol. 2022; 13:893160. 35620284 10.3389/fphar.2022.893160 PMC9127445

[45] Yoshihara K, Shahmoradgoli M, Martínez E, Vegesna R, Kim H, Torres-Garcia W, Treviño V, Shen H, Laird PW, Levine DA, Carter SL, Getz G, Stemke-Hale K, et al. Inferring tumour purity and stromal and immune cell admixture from expression data. Nat Commun. 2013; 4:2612. 10.1038/ncomms3612 24113773 PMC3826632

[46] McEligot AJ, Poynor V, Sharma R, Panangadan A. Logistic LASSO Regression for Dietary Intakes and Breast Cancer. Nutrients. 2020; 12:2652. 10.3390/nu12092652 32878103 PMC7551912

