# Supplementary material for: Integrative analysis to screen novel pyroptosis-related LncRNAs for predicting clinical outcome of glioma and validation in tumor tissue
Source: Aging (Albany NY). 2023 Mar 13;15(5):1628–51. doi: 10.18632/aging.204580 (PMC10042692; doi:10.18632/aging.204580)
Supplement: Supplementary Figures [file aging-15-204580-s001.pdf]

SUPPLEMENTARY FIGURES

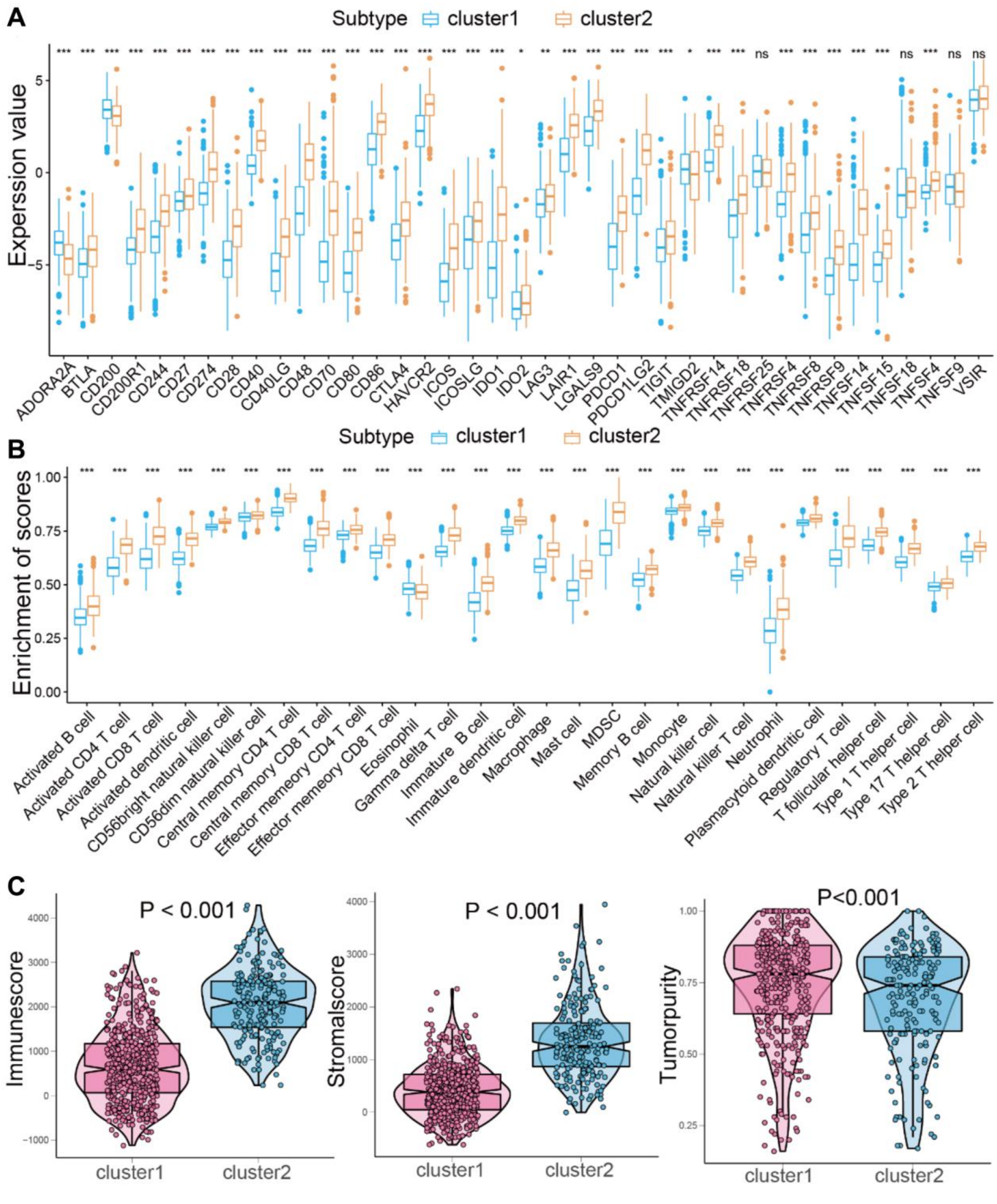

Supplementary Figure 1. The immune landscape of two cluster groups. (**A**) The expression value of immune checkpoint between cluster1 and cluster2 groups. (**B**) The enrichment scores of 22 kinds of immune cells between cluster1 and cluster2 groups. (**C**) The distribution of ESTIMATEscore in two cluster groups.

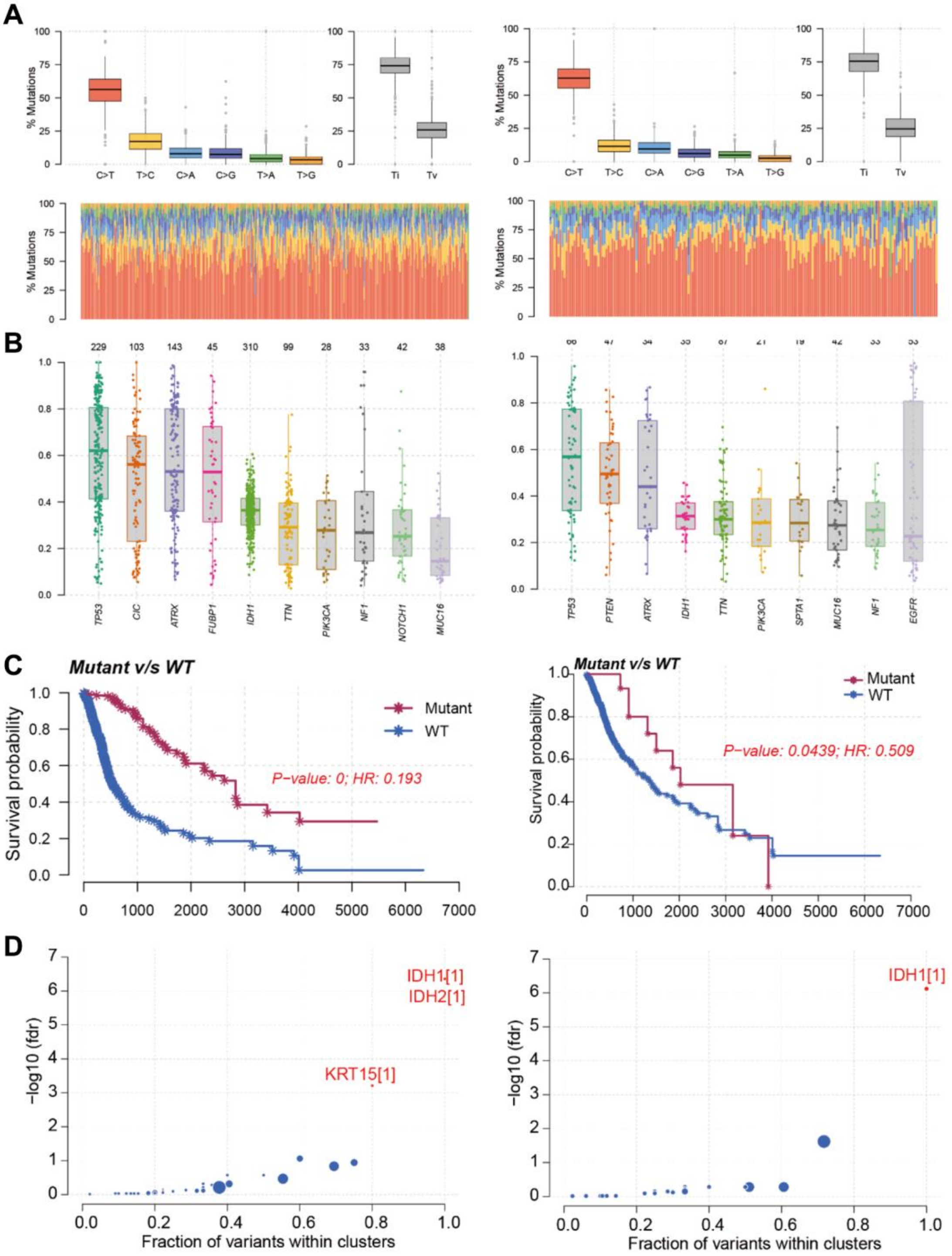

Supplementary Figure 2. Landscape of somatic mutation two cluster groups. (**A**) DNA substitution types including transition (Ti) and transversion (Tv). (**B**) Variant Allele Frequency expression of the top 10 genes of the two cluster groups. (**C**) Kaplan-Meier curves show the independent relevance between overall survival time and IDH1 mutation two cluster groups. (**D**) Distribution of tumor driver genes in two cluster groups.

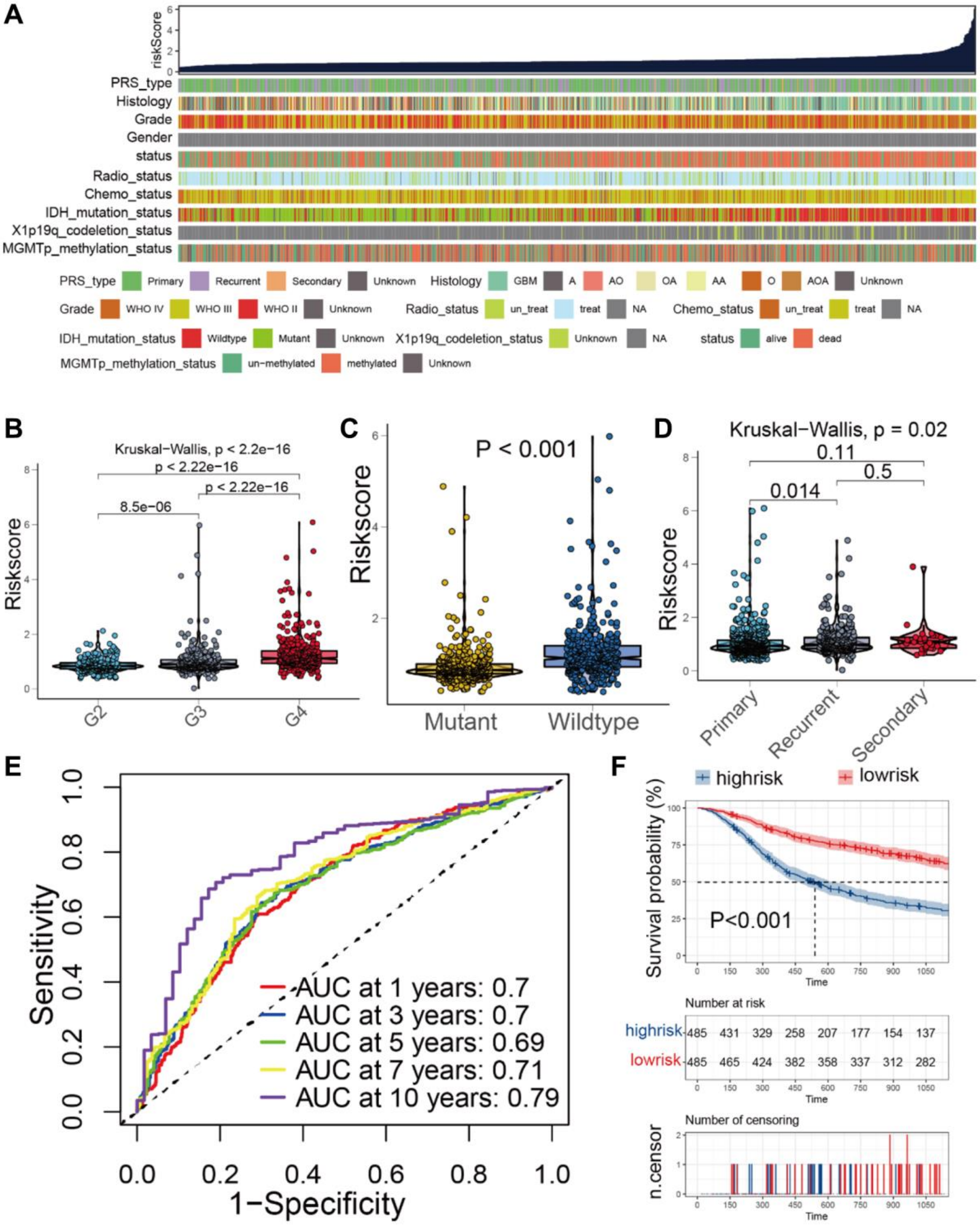

Supplementary Figure 3. The relationship between grades, IDH1 mutation types, 1p19q status, and riskscore in CGGA cohort. (**A**) An overview of the association between riskscore and clinical characteristics. (**B**–**D**) Analyses of the relationship between IDH1mut type, recurrence status, grades and PRLPM riskscore. (**E**) The timeROC curve to evaluate the prognostic value of PRLPM riskscore in CCGA cohort. (**F**) KM curve plot of OS for patients in high- and low-risk subgroups in CGGA cohort.

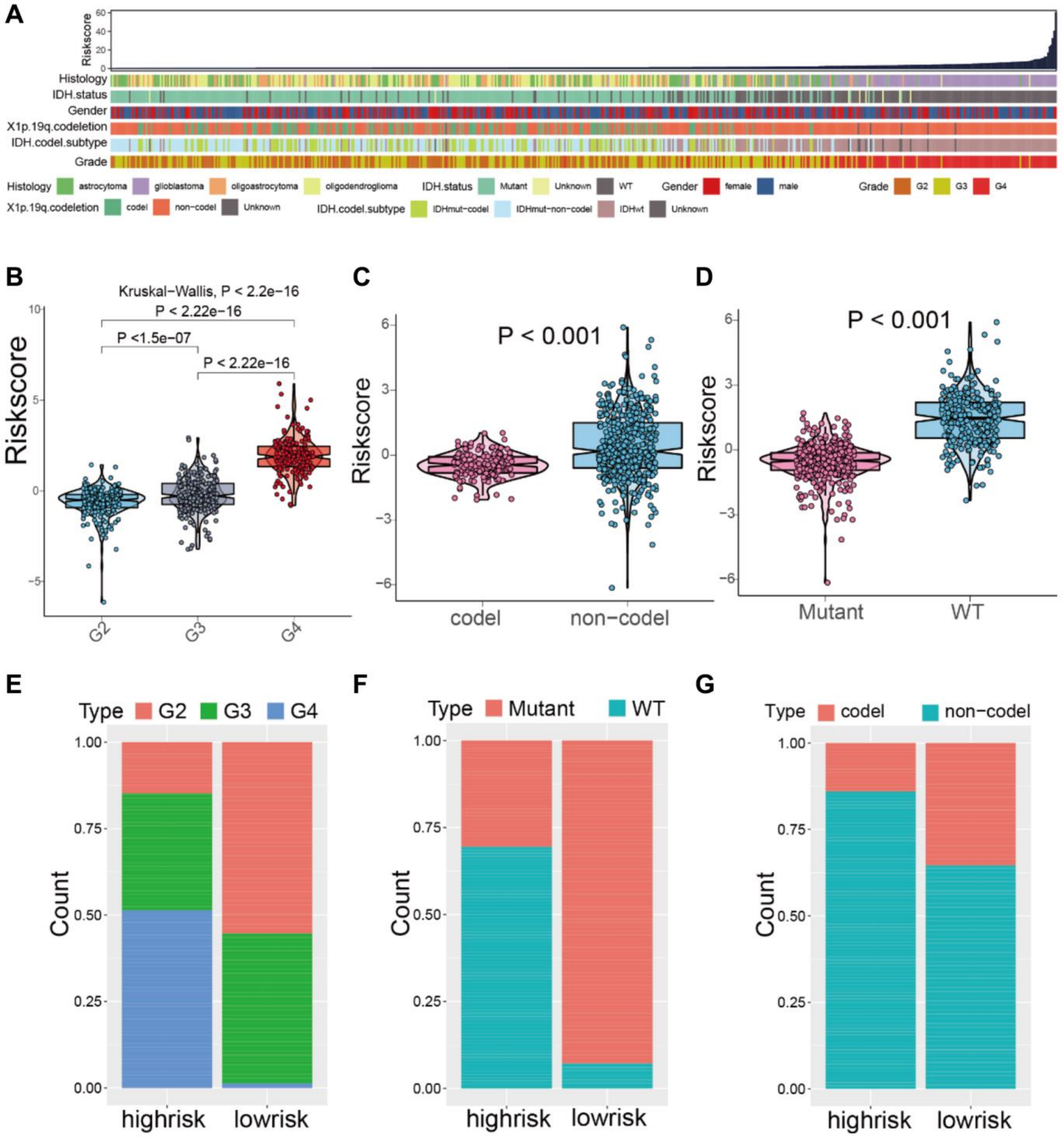

Supplementary Figure 4. The relationship between grades, IDH1 mutation types, 1p19q status, and riskscore in TCGA cohort. (**A**) An overview of the association between riskscore and clinical characteristics (Histology, IDH.status, Gender, IDHcodel.subtype and Grade). (**B**–**D**) Analyses of the relationship between IDH1 mutation type, recurrence status, 1p19q status and PRLPM riskscore. (**E**–**G**) The proportion of patients with IDH1 mutation type, recurrence status, 1p19q status in the high- or low-riskscore groups in the TCGA cohort.

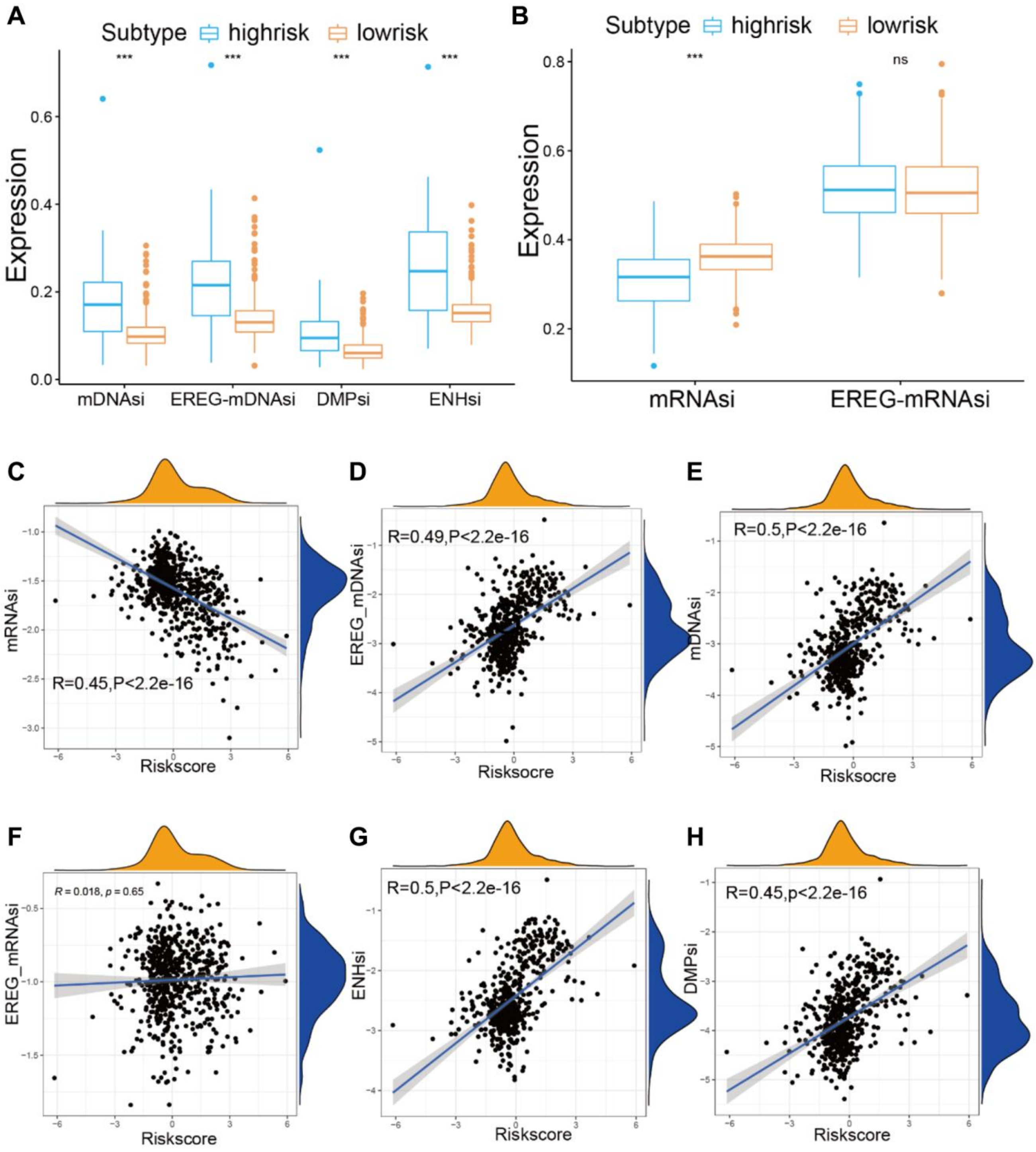

Supplementary Figure 5. Differences in stemness indices between the high- and low-risk groups. (**A, B**) Analyses of relationship between stemness indices and high- and low-risk groups. (**C**–**H**) The relationship between mDNAsi, DMPsi, EREG-mDNAsi, ENHsi, EREG-RNAsi, mRNAsi and riskscore.

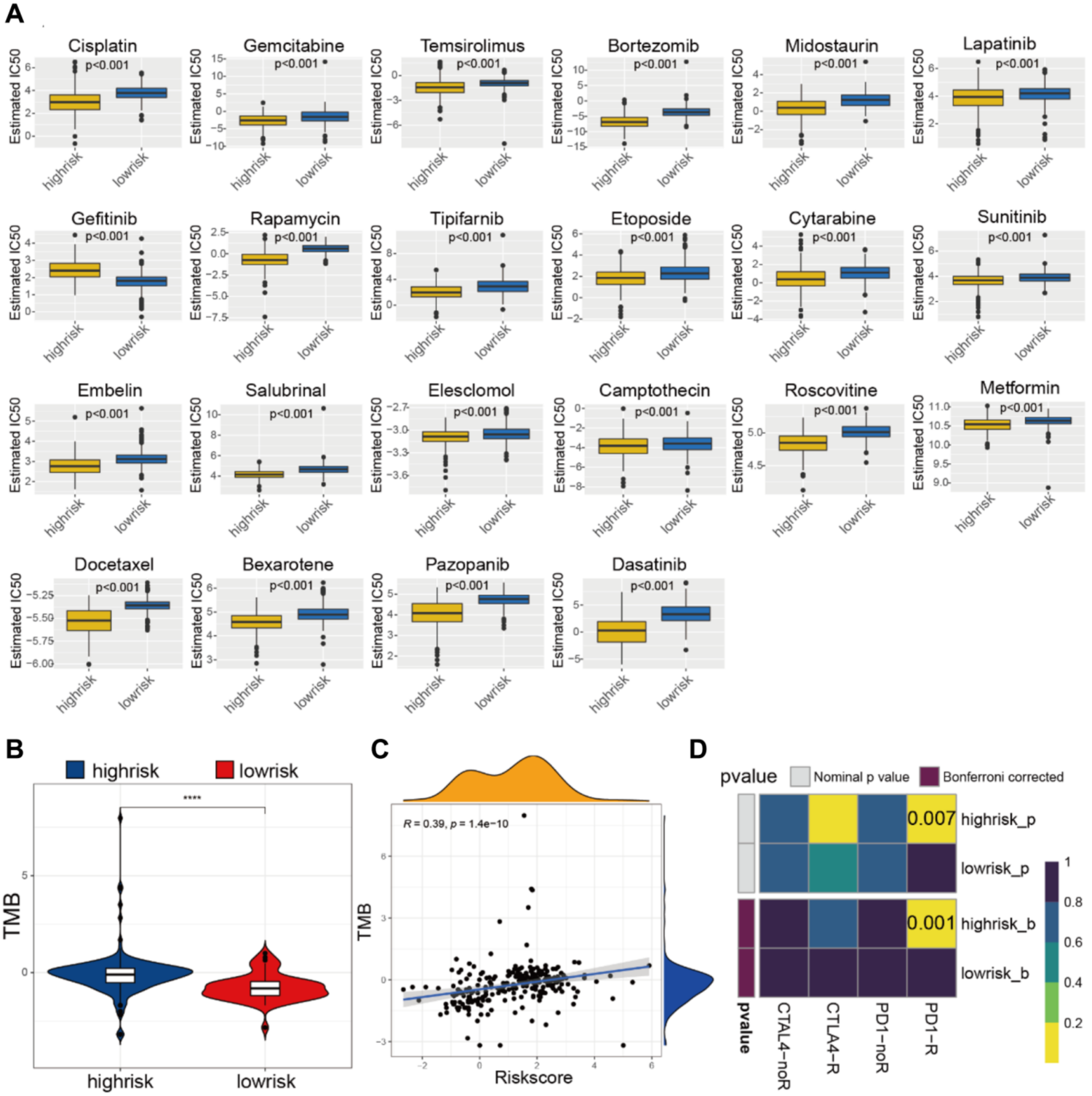

Supplementary Figure 6. Estimated drug sensitivity in patients with high- and low-risk groups. (**A**) The chemotherapy response of two prognostic subtypes for 30 common chemotherapy drugs. (**B**) The TMB expression value in high- and low-risk groups. (**C**) Correlations between the PRLPM riskscore and TMB for each cancer type. (**D**) Comparison of the effectiveness of PRLs signature-based stratification in predicting ICB responsiveness.

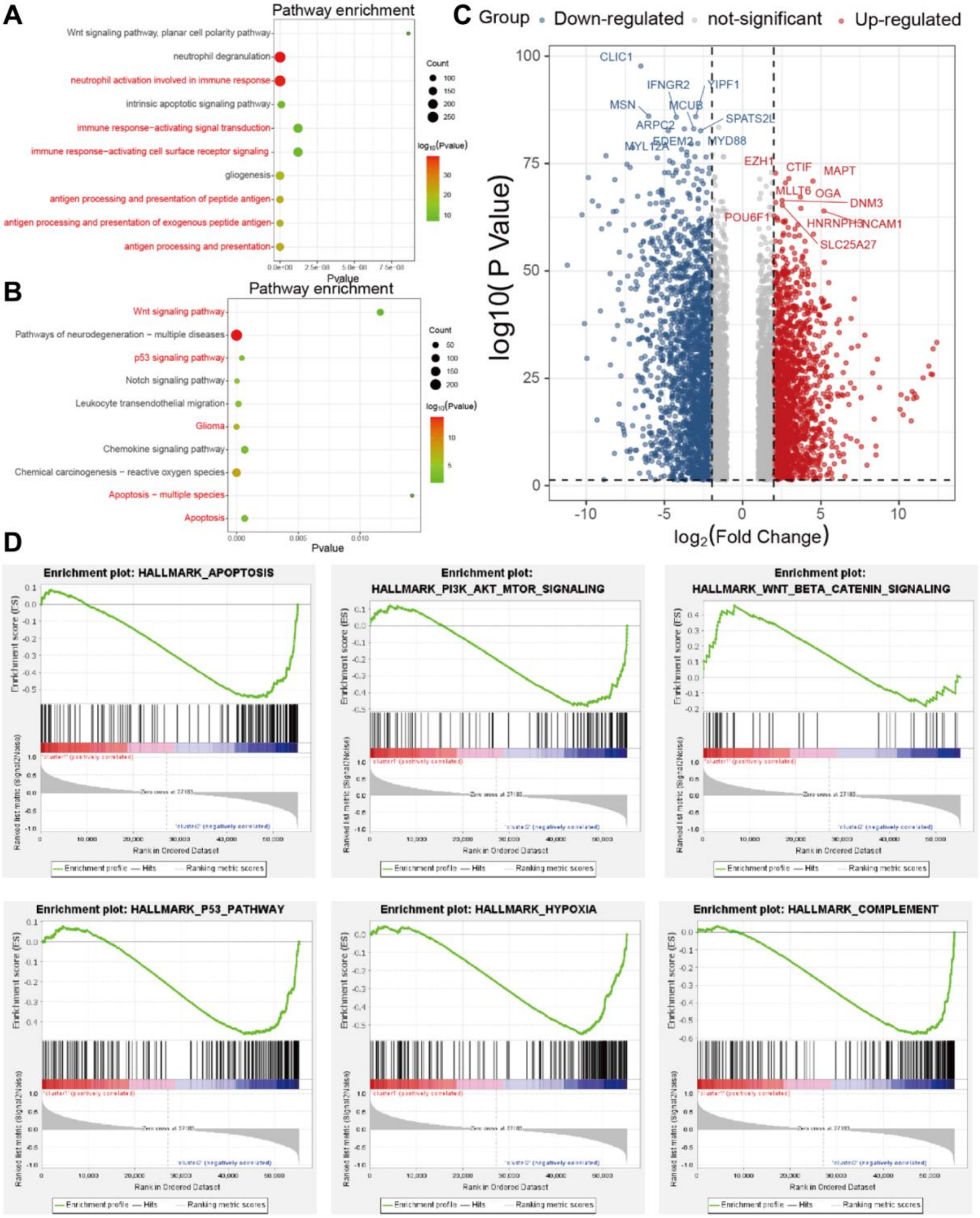

Supplementary Figure 7. Functional enrichment analyses of DEGs between high- and low-risk group in the TGCA cohort. (**A**, **B**) Gene Ontology (GO) and Kyoto Encyclopedia of Genes and Genomes (KEGG) enrichment analysis of DEGs. (**C**) The volcano plot for the DEGs between high- and low-risk group. (**D**) Gene set enrichment analysis (GSEA) enrichment analysis of DEGs.
